# Hope speech detection in Spanish: The LGBT case

**DOI:** 10.1007/s10579-023-09638-3

**Published:** 2023-03-17

**Authors:** Daniel García-Baena, Miguel Ángel García-Cumbreras, Salud María Jiménez-Zafra, José Antonio García-Díaz, Rafael Valencia-García

**Affiliations:** 1 I.E.S. San Juan de la Cruz, Jaén, Spain; 2 Computer Science Department, SINAI Research Group, CEATIC, Universidad de Jaén, Jaén, Spain; 3 Facultad de Informática, UMUTeam Research Group, Universidad de Murcia, Murcia, Spain

**Keywords:** Hope speech, Natural language processing, Language that relaxes hostile environments, Language that promotes equality, Diversity and inclusion

## Abstract

In recent years, systems have been developed to monitor online content and remove abusive, offensive or hateful content. Comments in online social media have been analyzed to find and stop the spread of negativity using methods such as hate speech detection, identification of offensive language or detection of abusive language. We define hope speech as the type of speech that is able to relax a hostile environment and that helps, gives suggestions and inspires for good to a number of people when they are in times of illness, stress, loneliness or depression. Detecting it automatically, in order to give greater diffusion to positive comments, can have a very significant effect when it comes to fighting against sexual or racial discrimination or when we intend to foster less bellicose environments. In this article we perform a complete study on hope speech, analyzing existing solutions and available resources. In addition, we have generated a quality resource, SpanishHopeEDI, a new Spanish Twitter dataset on LGBT community, and we have conducted some experiments that can serve as a baseline for further research.

## Introduction

Equality, Diversity and Inclusion (EDI) are important issues in all areas of the world. Language is a fundamental tool for communication and it must be inclusive and treat everyone equally. However, on social media this is not the case, as more and more offensive messages are posted towards people because of their race, color, ethnicity, gender, sexual orientation, nationality, or religion. As Chakravarthi (2020) stated, the importance of the social media in the lives of the members of vulnerable groups, such as people belonging to the Lesbian, Gay, Bisexual, and Transgender (LGBT) community, racial minorities or people with disabilities, has been studied and it has been found that the social media activities of a vulnerable individual play an essential role in shaping the individual’s personality and how he or she views society (Kitzie, 2018; Burnap et al., 2017; Milne et al., 2016). Moreover, it is a hot topic on social networks, in a multitude of languages. This paper focuses on research on the inclusion of people who belong to the LGBT community and it is approached on the study of *hope speech* as it can be used to promote positive content on social media, in pursuit of equality, diversity and inclusion.

Hope plays a crucial role in the well-being, recovery and restoration of human life (Chakravarthi, 2020). Greater hope is consistently related to better academic, athletic, physical health, psychological adjustment, and psychotherapy outcomes. Hope theory is comparable to theories of learned optimism, optimism, self-efficacy, and self-esteem (Snyder, 2002); and can be subdivided into four categories:

1. *Goals*, which are evaluable and uncertain. They are described as the anchors of hope theory, as they provide a direction and endpoint for hopeful thinking (Snyder, 1994, 2000).
2. The *pathway thoughts*, which refer to the paths we take to reach our desired goals and the individual’s perceived ability to produce these pathways (Snyder, 2000).
3. *Agency thoughts*, which refer to the motivation we have to undertake the paths towards our goals and our perception of our own level of agency, that is call self-efficacy (Snyder, 2000).
4. *Barriers*, which block the achievement of our goals. In the event of a barrier, we can either give it up or use our thought paths to create new paths.

**Fig. 1:**
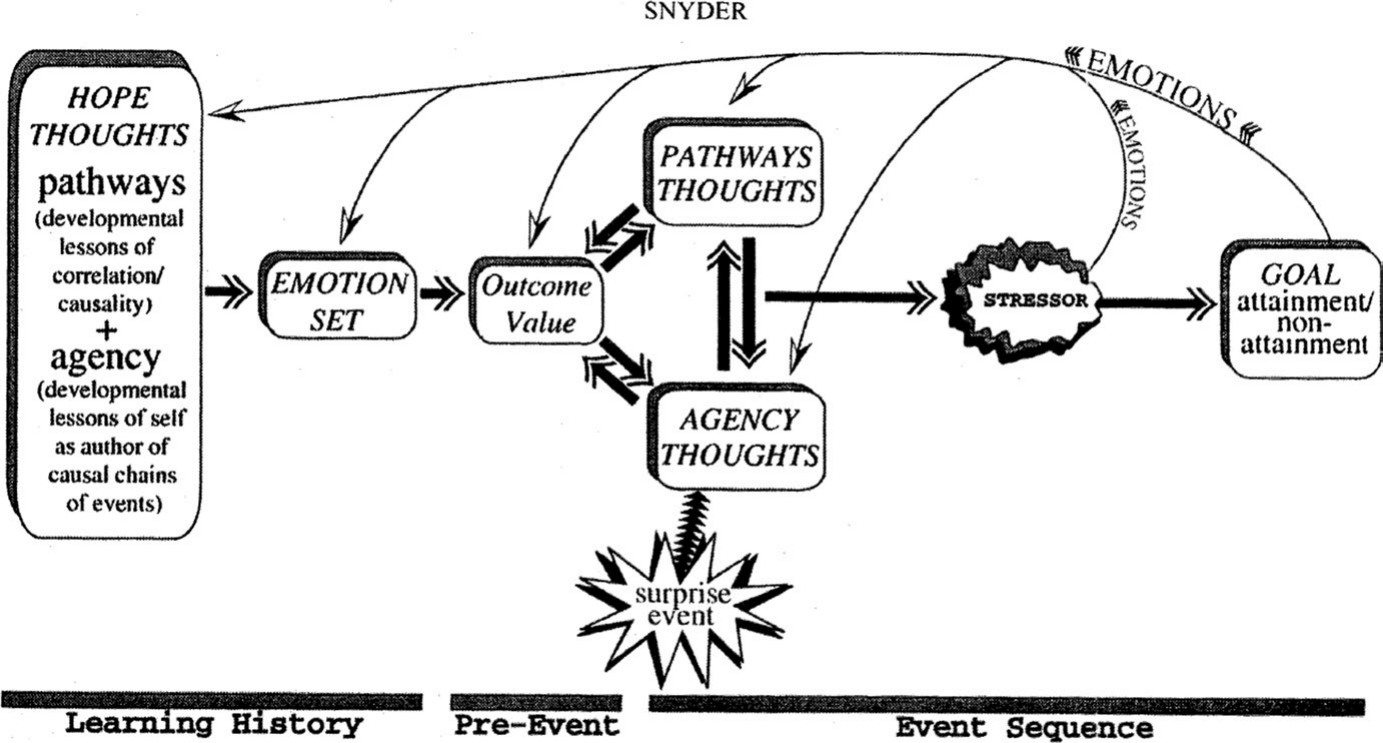
Snyder, “Hope Theory: Rainbows in the Mind”

As shown in Figure 1, Snyder sees hope as a cognitive process where we first imagine a future goal, then decide if it is valuable enough and, if barriers are not too big, finally we conclude to pursue that goal. Thus, with this aim, we discern whether pathways exist that might get us there, evaluate our past history of successes and failures in goal attempts, and reflect on the level of agency or motivation we feel regarding our ability to see this hope through to an eventual goal attainment.

Individuals with high hope do not react in the same way to barriers as individuals with low hope, but instead view barriers as challenges to overcome and use their pathway thoughts to plan an alternative route to their goals (Snyder, 1994, 2000). In addition, high hope has been found to correlate with a number of beneficial elements, such as academic performance (Snyder et al., 2002) and lower levels of depression (Snyder et al., 1997). In contrast, low hope is associated with negative outcomes, such as reduced well-being (Diener, 2009).

As described by Chakravarthi (2020), in this work we consider hope speech those comments/posts that promote the values of equality, diversity and inclusion. We provide some examples below:

**Table:** 

Las mujeres trasn son mujeres. Los hombres trasn son hombres. Las personas LGBTQ+ tienen derecho a la identidad y a la familia, a vivir libres de acoso y de discriminación. Todas las personas son iguales en libertad y derechos por el solo hecho de haber nacido. #OrgulloLGTBI	
*Trans women are women. Trans men are men. LGBTQ+ people have the right to identity and to form a family, the right to live free from harassment and discrimination. All human beings are born equal in dignity and rights. #LGTBIPride*

**Table:** 

Feliz dia del orgullo, mostremos los orgullosos que estamos de ser nosotros #pride #Orgullo2021 #OrgulloLGTBI	
*Happy Gay pride, let’s show how proud we are of being ourselves**#pride #2021Pride #LGTBIPride*	

This paper analyzes the state of the art of automated *hope speech* detection technologies from the perspective of Natural Language Processing (NLP). Automated detection of *hope speech* can be especially useful in promoting the dissemination of hopeful messages to those in difficult times and can be used to promote positive messages to support EDI. Previous studies have shown that a snowball effect occurs in social media, i.e., abusive comments lead to more abusive comments and positive comments inspire people to leave more positive comments (Sundar et al., 2022; Muchnik et al., 2013). Facebook conducted an experiment by modifying its “Newsfeed” algorithm to show more positive or negative posts to certain users (Kramer et al., 2014). Their results showed that people tend to write positive posts when they see happy posts in their newsfeed and vice versa. Therefore, it is important to reinforce positivity on social media by focusing on *hope speech*.

The main contributions of this work are:

1. Theoretical study of the concept *hope speech*, as well as its treatment from the NLP point of view.
2. Analysis of the existing solutions and discussion of the problems derived from them.
3. Review of available resources, providing experiences and an accessible introduction to those researchers who may be interested in solving this problem.
4. A new dataset about the LGBT community for Spanish *hope speech* detection, created using Twitter as a source of information.
5. Baseline experiments with some Machine Learning (ML) and Deep Learning (DL) algorithms, including transformers.
6. Error analysis to determine future directions of the study.

The rest of the paper is organized as follows. Section 2 defines the term *hope speech*, shows its relationship and differences with the term *hate speech*, and presents the problems solved by its automated detection. Section 3 reviews the existing datasets and the experiments conducted on them. Section 4 presents a new dataset for *hope speech* detection to promote LGBT inclusion, baseline experiments and error analysis. Finally, Section 5 summarizes the conclusions of this study and shows future work.

## Hope speech and previous works

*Hope speech* is the type of speech that is able to relax a hostile environment (Palakodety et al., 2019) and that helps, gives suggestions and inspires for good to a number of people when they are in times of illness, stress, loneliness or depression (Chakravarthi, 2020). Detect it automatically, so that positive comments can be more widely disseminated, can have a very significant effect when it comes to combating sexual or racial discrimination or when we seek to foster less bellicose environments (Palakodety et al., 2019).

As stated in the work of Chakravarthi (2020), *hope speech* is defined as the language that is related to fostering individuals’ potential, supporting them and reaffirming their self-confidence, as well as, again, making motivational and inspirational suggestions in difficult times of illness, loneliness, stress or depression (Snyder et al., 2003).

However, Palakodety et al. (2019) differ from the above definition and establish as *hope speech* simply that which has the capacity to relax situations of tension and violence. Even, Chakravarthi (2020) also introduces a possible variation of what is meant by *hope speech*, now taking into account the ability of language to promote equality, diversity and inclusion of women belonging to the fields of science, technology, engineering and management (STEM), lesbian, gay, bisexual, transgender, intersex, and queer individuals, and racial minorities and individuals with disabilities.

### The importance of context

In this regard, it is clear that the definition of *hope speech* is not clear and depends entirely on the context of the texts. For example, while in the work of Chakravarthi (2020) the definition has to do with promoting diversity, equality and inclusion, none of this is necessary in the work of Palakodety et al. (2019), where they focus on relaxing situations of tension and violence. Therefore, to approach this task it will be necessary to carefully choose a definition of this term and based on this definition of *hope speech*, for example, correct annotation guidelines will be established, with which resources such as datasets can be generated, and related experiments can be carried out.

Some examples of *hope speech* messages are as follows:

- “We will survive these things.”. In this case, Chakravarthi (2020) refers to a type of speech that gives hope on the future.
- “Say no to any war. We need a world without war.” This time, in the work of Palakodety et al. (2019), a type of text that promotes peace is discussed.

### Our definition and characterization of hope speech

According to the above definitions and for the specific context of promoting diversity, equality and inclusion, we consider a text as *hope speech* if it explicitly supports the social integration of minorities, is a positive inspiration for the LGBT community, explicitly encourages LGBT people who might find themselves in a situation or unconditionally promotes tolerance.

### Why is hope speech detection relevant?

Psychology has extensively studied the positive influence of hope on goal attainment. Chang (1998) analyzes the results obtained by a set of students with different levels of hope and shows that those who have a greater conviction regarding the possibility of achieving their academic goals also end up having better results. This study shows that students with a higher level of hope tend to develop better problem-solving strategies, and even claims that hope is a very important predictor of students’ personal satisfaction. Likewise, the absence of hope can affect health. In summary, this work validates what has been proposed in previous works (Snyder et al., 1991, 2003) and asserts that:

1. Students with greater hope toward goal attainment end up developing better coping skills.
2. Such subjects also tend to solve problems driven more by logical reasoning and less by emotional reasoning.
3. Hope has a very important direct impact on a student’s quality of life.

In part due to the success of studies such as those just cited, positive psychology has continued to conduct research, and has even developed a branch focused on the influence it has on the work environment, which has been called: *positive organizational behavior* (or from its acronym, *POB*).

Youssef and Luthans (2007) present a study similar to that of Chang (1998), but this time focusing on workers rather than students. The results of Youssef and Luthans (2007) show that, despite the fact that positive psychology has been little studied in work environments, the influence of *POB* on workers’ performance, satisfaction and happiness, as well as on the achievement of goals, is very big and, therefore, workers who show a greater hope for improvement in their company tend to have an especially positive influence on all the aforementioned aspects.

Cover (2013) studies the influence of a digital tool such as the web: *It Gets Better*^1^, with a view to helping young people from the *LGBT* collective to get out of stressful situations derived from bullying by fostering hope and resilience. Studies such as the aforementioned one extend what was seen in previous works (Snyder et al., 1991; Chang, 1998; Snyder et al., 2003) and underline the importance of *hope speech* in order to positively influence people who are going through emotionally difficult situations. Next, it can be found an example of this type of hope speech extracted from our new dataset:

**Table:** 

Se conmemora la revuelta de Stonewall, dando lugar a un movimiento revolucionario en contra de la discriminación y abusos. El closet es para la ropa. Seamos más libres y que reine en el pueblo el amor y la igualdad #Orgullo #OrgulloLGTBI #LoveIsLove	
*Today we celebrate the Stonewall riots, the birth of a revolutionary movement against sex discrimination and sexual harassment. Closets are for clothes. Let’s be free and long live to love and gender equality* *#Pride #LGTBIPride #LoveIsLove*	

On the other hand, *hope speech* has also been proposed for the protection of freedom of expression, as the deletion of content related to *hate speech* is considered in some studies against freedom of speech (Chakravarthi, 2020). There are many works where automated moderation tools are studied to remove offensive, abusive or any other type of *hate speech* comments. One of the most relevant is that of Chandrasekharan et al. (2017), which reviews the consequences of the shutdowns of numerous popular Reddit channels or communities because they systematically violated the abuse prevention policies published by the platform. However, the authors concluded that the effectiveness that the closure of these channels had in ensuring compliance with the platform’s rules is uncertain, as it is quite likely that users of these sections will continue to engage in such behavior on other channels because their freedom of expression was being deprived. One of the most commonly used strategies to avoid having to delete posts with hostile content is the use of counter-narratives, which consists of publishing texts that refute what has been stated in comments with inaccurate information. Although this strategy does take into consideration the importance of preserving the right to freedom of expression, as Mathew et al. (2019) points out, intervening directly in a conversation using *counter-narratives* usually leads to an escalation of hostility, even though we can see it as something positive for the person responsible for the publication to know why their comments have required moderation. Finally, in the work of Palakodety et al. (2019), and taking into account all the factors mentioned above, it is shown that in news videos published on YouTube about the hostilities between India and Pakistan, regarding the Kashmir region, the *hope speech* comments contributed to relax tensions and were positive for the refugees of the Rohinyá minority. In this way, it seems that the promotion of *hope speech* is also positive for the protection of freedom of expression.

### Hope speech vs. hate speech

We can define *hate speech* as a particular form of offensive language that makes use of stereotypes to express an ideology of hate, or as any communication that disparages a person or group on the basis of some characteristic such as race, color, ethnicity, gender, sexual orientation, nationality, religion or other aspect (Warner and Hirschberg, 2012). In recent years, it is one of the most widely discussed research topics in the Natural Language Processing research community. Hate speech is present in our corpus too and an example can be seen below:

**Table:** 

Todo un mes dedicando medio telediario a las #LGTBI de los c0h0nes. Vete al carajo @user !! *A whole month talking about the fucking #LGTBI people. @user, go fuck off !!*	

Although the detection of *hate speech* and *hope speech* are related classification problems, the language used in both is different (hate vs. hope). This creates the need to study them separately, since the fact that a message does not show hate does not imply that it denotes hope. In our experience with these two tasks, the level of difficulty is similar, although *hate speech* detection is a more advanced task in the scientific community and, consequently, more resources have been generated and more experimental work has been done. The use of hate speech in social networks can have negative psychological effects on users, even leading them to extreme cases. We believe that the detection of *hope speech* could be useful to encourage people at times when they need it and thus try to reduce cases of depression and suicide. In addition, there is a need to promote positive content on social media in pursuit of equality, diversity and inclusion.

## Previous datasets and experiments

This section presents existing datasets (see Sect 3.1), the results obtained in previous works (see Sect. 3.2) and a discussion thereof (see Sect. 3.3). As this is a recent task to be tackled automatically from NLP, there are available only a few corpus.

### Previous datasets

#### HopeEDI

The HopeEDI dataset (Chakravarthi, 2020) contains comments in English, Tamil and Malayalam. It consists of data obtained from comments posted on *YouTube* videos that were collected from November 2019 to June 2020 using the open source tool *YouTube* *Comment Scraper*^2^. The corpus can be downloaded free of charge at Hugging Face^3^.

The subject matter of the comments written in English are *EDI*, including women in the *STEM* group and people from the *LGBT* collective, COVID-19, the *Black Lives Matters* movement, UK versus China, US versus China, and Australia versus China. The comments come from videos posted by citizens from English-speaking countries such as Australia, Canada, Ireland, the United Kingdom, the United States and New Zealand. Topics chosen for comments in Tamil and Malayalam are *LGBT*, COVID-19, presence of women in the *STEM* group, Sino-Indian war and conflicts related to Dravidian peoples. In this case, the comments come from videos posted by Indian and Sri Lankan users. It is important to note that since India is a multilingual country, many of the comments may be written in several languages at the same time (*code-mixing*).

**Table 1:** HopeEDI dataset statistics

	English	Malayan	Tamil
Distribution of data by class			
* Hope_speech*	2484	2052	7899
* Non_hope_speech*	25,940	7765	9816
Other language	27	888	2483
Total	28,451	10,705	20,198
Training-development-test distribution			
Training	22,762	8564	16,160
Development	2843	1070	2018
Test	2846	1071	2020
Total	28,451	20,198	10,705

The HopeEDI corpus has a total of 59,354 entries, of which 28,451 are written in English, 20,198 in Tamil and 10,705 in Malayalam. The entire corpus was fragmented so that 80% was used for training, 10% for development and the remaining 10% for testing. The corpus statistics by class and set can be seen in Table 1.

The distribution of comments labeled as *Hope_speech*, *Non_hope_speech*, and *Other language* (for comments that were not in the required language^4^) is very uneven and the corpus is not balanced. For example, in the case of English writings about 8% correspond to *Hope_speech*, 91% to *Non_hope_speech* and the remaining 1% (namely twenty-seven texts) to *Other language*. For the case of Tamil, the distribution is more equitable, but the author admits to having had problems related to *code-mixing* and the data provided may not be reliable. Considering all three languages at the same time, the approximate final distribution would be 21% of comments labeled as *Hope_speech*, 73% as *Non_hope_speech* and 4% as *Other language*.

#### India-Pakistan

This dataset contains data from English comments posted on videos from YouTube (Palakodety et al., 2019). The researchers chose this site as the source of the data because it is the most widely used video broadcasting platform in India and Pakistan today.

For their compilation, a series of queries were prepared and then extended with searches related to the Kashmir conflict by consulting trends from India and Pakistan that took place between February 14, 2019 and March 13, 2019. Finally, such queries were used to search for related videos on *YouTube* and subsequently obtain their comments using the public API of that social network.

The comments are all written in English and come from mainly Indian and Pakistani users. There are also comments submitted by immigrants from India and Pakistan, whose were in Bangladesh, Nepal, United States, United Kingdom, Afghanistan, China, Canada and Russia. In this case, the origin of the users was taken into account with the intention of maintaining an equal representation of citizens belonging to both sides of the conflict.

The training set consists of 2,277 hope speech and 7,716 non-hope speech comments. For validation and test, the authors did not share the exact numbers but published that they used an 80/10/10 distribution. Therefore, the validation and test numbers in Table 2 are approximate. Unfortunately, this corpus it is not publicly available.

**Table 2:** India-Pakistan dataset statistics

	Hope speech	Non-hope speech
Training	2277	7716
Development	285	965
Test	285	965
Total	2847	9646

#### KanHope

KanHope dataset (Hande et al., 2021) contains comments in code-mixed Kannada-English. All data was collected with the app YouTube Comment Scraper between February 2020 and August 2020. The dataset is publicly available on Hugging Face^5^.

KanHope gathers comments from several videos on distinctive topics such as movie trailers, India-China border dispute, people’s opinion about the ban on several mobile apps in India, Mahabharata, and other social issues that involved oppression, marginalization, and mental health. KanHope dataset authors emphasize on the inclusion of people of marginalized communities, such as Lesbian, Gay, Bisexual, Transgender, Intersex, and Queer, Questioning (LGBTQ) communities, racial and gender minorities. All comments were from users based in India and, being it a multilingual country, researchers were motivated to extract the comments to work on code-mixed texts.

This dataset has a total of 6176 entries and the entire corpus was fragmented so that 80% was used for training, 10% for development, and 10% for testing. Comments were labeled as *Hope Speech* and *Non_hope Speech*. The classes are not equally distributed in the dataset and while *Non_hope Speech* accounts for 65.81%, *Hope Speech* accounts for 34.19%. The number of comments in each category for the training, development, and test sets can be found in Table 3.

**Table 3:** KanHope dataset statistics

	Hope speech	Non_hope speech
Training	1675	3265
Development	227	391
Test	210	408
Total	2112	4064

### Experiments and results

The following are the experiments carried out by the authors of the existing datasets: HopeEDI, India-Pakistan, and KanHope. A summary of the results obtained is presented in Table 4.

#### HopeEDI

For the HopeEDI corpus, its author applied different machine learning algorithms on a TF-IDF (*Term Frequency-Inverse Document Frequency*) representation of the tokens (Chakravarthi, 2020). In all cases, the *scikit-learn* library was used to create the classifiers. Specifically, the corpus was evaluated with the following: Bayesian multinomial classifier (*multinomial Naïve Bayes* or MNB) with a value of *alpha* equal to 0.7, k-nearest neighbors method, support vector machine (SVM), decision tree (DT), and with Logistic Regression (LR). The development set was used to perform fine tuning of the models from which the results of the experiments were evaluated.

As for the results obtained, the author said that, in general, they were not at all as desired. DT performed better in English and Malayan than SVM, MNB, KNN, and LR; but worst than LR in Tamil. In any case, for all commented techniques, results scored an F1 value no better than 0.56 and, consequently, they were quite disappointing.

This corpus was also used in the Shared Task on Hope Speech Detection for Equality, Diversity, and Inclusion (Chakravarthi and Muralidaran, 2021). A large proportion of the participants outperformed the results provided as a baseline by the authors of the corpus, which were discussed in the previous paragraph. Most of the competitors explored deep learning algorithms. The top F1-scores were 0.61 (Sharma and Arora, 2021), 0.85 (Hossain et al., 2021) and 0.93 (Mahajan et al., 2021) for Tamil, Malayalam and English respectively.

#### India-Pakistan

For the India-Pakistan corpus (Palakodety et al., 2019), the authors used a logistic regression with L2 regularization classifier (*Ridge Regression*). The experiment was run a total of one hundred times on one hundred random sections of the dataset, always respecting the 80/10/10 distribution discussed above. The features used were the following:

- N-grams up to size 3.
- A sentiment value that adds one to phrases *hope speech*, subtracts one from phrases *hate speech* and neither adds nor subtracts any value to the rest. For this purpose, authors created a comprehensive set of intent phrases.
- 100-dimensional polyglot word-embeddings. They were calculated by fastText using the skip-gram model.

In this case, the classifier achieved an F1 value of 0.79, while a popular model, such as Stanford CoreNLP, was only able to obtain an F1 of 0.33. Although the CoreNLP system is not designed to classify hope but the sentiment of texts, the authors of the corpus used it to show that the task of detecting *hope speech* is different from simple sentiment analysis and therefore requires a specific approach.

In addition, the researchers tested the classifier with another thousand comments that were not in the dataset and obtained results of 0.85 for the precision value and 0.98 for completeness (*recall*). Therefore, from the above values, they obtained an F1 value of 0.91 for new comments that had not been included in the dataset.

#### KanHope

The KanHope corpus authors (Hande et al., 2021) applied from primitive machine learning to complex deep learning approaches. They used scikit-learn library for data processing and to implement the machine learning algorithms. Hugging Face pretrained language models were used for transformers. The input features used were the Term Frequency Inverse Document Frequency (TF-IDF) values of up to 5 grams. In this case, the corpus was evaluated with Logistic Regression, k-nearest neighbors (KNN) algorithm, decision tree (DT), random forest (RF), and multinomial Naive Bayes (MNB), in relation to machine learning alternatives, and with the next fine-tuned pretrained language models: BERT, Multilingual-BERT (mBERT), RoBERTa, XLM-RoBERTa (XML-R), and a dual-channel language model based on the architecture of BERT that the authors called DC-BERT4HOPE, this latter is the result of fine-tuning a language model based on BERT with the code-mixed data and its Google Translate API English translation.

As exposed in Table 4, the model DC-BERT4HOPE (roberta-mbert) obtained the best results for F1-scores with 0.752, followed by DC-BERT4HOPE (bert-mbert): 0.735, mBERT: 0.726, DC-BERT4HOPE (roberta-xlm): 0.720, and random forest with 0.706.

**Table 4:** Results of benchmark experiments conducted by the authors of the HopeEDI, India-Pakistan, and KanHope corporas (*NA: Not Applicable, **NP: Not Provided)

	F1			
	English	Kannada	Malayan	Tamil
HopeEDI				
LR	0.450	NA	0.530	0.550
DT	0.460	NA	0.560	0.510
ULMFiT	NP*	NA	NP	**0.610**
XLM-R	**0.930**	NA	**0.850**	0.600
RoBERTa	**0.930**	NA	NP	NP
India-Pakistan				
LR	**0.790**	NA	NA	NA
KanHope				
LR	NA	0.634	NA	NA
BERT	NA	0.702	NA	NA
DC-BERT4HOPE (roberta-xlm)	NA	0.702	NA	NA
mBERT	NA	0.726	NA	NA
DC-BERT4HOPE (bert-mbert)	NA	0.735	NA	NA
DC-BERT4HOPE (roberta-mbert)	NA	**0.752**	NA	NA

### Discussion

The HopeEDI author attributes the poor results obtained in his experimentation to a class imbalance problem and highlight the importance of automatically detecting hope speech to encourage positivity and induce compassion and acceptable social behavior. Participants in the shared task improved on the author’s results with the use of deep learning techniques, obtaining a top F1-score of 0.93. His roadmap includes extending the study by introducing a larger dataset with further fine-grained classification and content analysis.

On the other hand, India-Pakistan researchers manifest to be satisfied with their results, and as in HopeEDI and KanHope, underline the importance of detecting hostility-defusing content as it is *hope speech*. The authors of this dataset studied the effect of *hope speech* comments in war and peace intents and found a correlation between hostility-defusing and *hope speech* heavy usage.

In addition, KanHope researchers highlight again the need to motivate positivity and *hope speech* in platforms to instigate compassion and assert reassurance. In relation to performance, even though this dataset also has class imbalance problems, the authors obtained good F1-scores using deep learning strategies.

Consequently, given the importance of reinforcing positivity in social media to support EDI and due to the lack of a Spanish dataset to develop automatic systems for hope speech detection, we have created SpanishHopeEDI. Specifically, we have extended the HopeEDI dataset with a balanced corpus in Spanish as HopeEDI author suggests in his work. Our dataset, as HopeEDI and KanHope, is about equality, diversity, and inclusion, but focuses on LGBT. Finally, it should be mentioned that our SpanishHopeEDI dataset has been included in the second workshop on Language Technology for Equality, Diversity and Inclusion that was held as a part of the ACL 2022 (see Sect. 4.4).

## SpanishHopeEDI: A new Spanish dataset and baseline experiments

In this section we present the compilation of SpanishHopeEDI, a new dataset for *hope speech* detection in Spanish, as well as several baseline experiments we have performed.

### A new *hope speech* Spanish dataset

The SpanishHopeEDI dataset consists of 1,650 LGBT-related tweets annotated as HS (Hope Speech) or NHS (Non Hope Speech). A tweet is considered as HS if the text: (i) explicitly supports the social integration of minorities; (ii) is a positive inspiration for the LGTBI community; (iii) explicitly encourages LGTBI people who might find themselves in a situation; or (iv) unconditionally promotes tolerance. On the contrary, a tweet is marked as NHS if the text: (i) expresses negative sentiment towards the LGTBI community; (ii) explicitly seeks violence; or (iii) uses gender-based insults. The following is a description of the collection and annotation process carried out for its generation.

### Data collection

The dataset was created from LGBT-related tweets. It consists of Spanish tweets that were collected using the Twitter API (June 27, 2021 to July 26, 2021). As seed for the search we used a lexicon of LGBT-related terms, such as #OrgulloLGTBI and #LGTB. The corpus comprised 3,200 tweets, which were eligible for manual annotation. We discarded tweets of less than five words to get an unambiguous context. Table 5 shows the dataset statistics.

**Table 5:** Dataset statistics

Statistics	
Number of words	60,058
Vocabulary size	12,018
Number of tweets	1650
Number of sentences	2886
Average number of words per sentence	21
Average number of sentences per tweet	2
Distribution of data by class	
HS	825
NHS	825
Total	1650
Training-development-test distribution	
Training	990
Development	330
Test	330
Total	1650

### Data annotation and quality

Two PhD researchers and one PhD student conducted the annotation process. Each tweet was annotated with one of the following labels: HS (Hope Speech), NHS (Non Hope Speech) and None (other).

In a first stage, the three annotators elaborated an annotation guide to establish the conditions for this labeling^6^. A first classification of 200 random tweets was performed by all the annotators and the results were reviewed. The conclusion was that several tweets could not be labeled as either HS or NHS, and more cases were added to the None class. After several tagging tests and further discussion, we defined these labeling rules:

- A tweet is marked as HS if the text:
  1. explicitly supports the social integration of minorities.
  2. is a positive inspiration for the LGBT community.
  3. explicitly encourages LGBT people who might find themselves in a situation.
  4. unconditionally promotes tolerance.
- A tweet is marked as NHS if the text:
  1. does not express any positive sentiment towards the LGBT community.
  2. (HateSpeech) explicitly seeks violence.
  3. (HateSpeech) uses gender-based insults.
- In any other case, for example if the tweet is a fact or if it does not express any opinion on the matter, it is marked as None.

In the overall labeling process, each tweet was annotated by at least two annotators, with the third annotator intervening when there were discrepancies in the final class. Each annotator classified a total of 2,000 tweets.

We evaluated the inter-annotator agreement to ensure the quality of the annotation using the coding reliability (krippendorff k,, 2011) and Cohen’s kappa (Cohen, 1960) scores. Krippendorff’s Alpha inter-coder reliability of 88.1% and Cohen’s Kappa score of 0.87 reflect the quality of the corpus.

Regarding the limitations found in the annotation process, due to the complexity and inaccurate nature of the hope speech definition, it has often been necessary to double check the annotation guidelines, review several examples, and discuss with the other annotators about some mismatched labels in order to finally be able to find the correct categories for all tweets. Therefore, the annotation guide has been tweaked several times in an effort to minimize any potential mistakes. It can be found at the link provided in the Availability of data and material section.

Below there are a couple of examples of tweets labeled as HS and NHS, respectively.

**Table:** 

HS: Que homosexuales, trans, etc... No puedan ir tranquilos por la calle es algo inadmisible, lucho por un dia sin discriminaciones por ser quien tu eres. #justiciaparasamuel #LGTB	
*That homosexuals, trans, etc... Can not go quietly down the street is something inadmissible, I fight for a day without discrimination for being who you are. #justiciaparasamuel #LGTBI*

**Table:** 

NHS: Lo asesinaron por un movil gilipollas! apenas se ha sabido quienes fueron 7 de los que le dieron la paliza como para que se supiese que era por ser gay. El colectivo lgtbi+++ y la izquierda como siempre haciendo politica de las desgracias de los demas, menuda escoria que soys.	
*They killed him for a cell phone, assholes! It has hardly been known who were 7 of those who beat him up so that it was known that it was because he was gay. The lgtbi+++ collective and the left are making politics out of the misfortunes of others as always, what scum you are.*	

Table 6 shows the original dataset statistics. As it can be seen, 825 tweets were annotated as HS, 1,301 as NHS, and 1,062 as None. Subsequently, a balanced dataset was generated, containing the 825 tweets labeled as Hope Speech, and 825 random tweets labeled and validated as Non Hope Speech, making a total of 1650 tweets (see Table 7). This is the dataset used in the experiments and included in the Shared Task on Hope Speech Detection for Equality, Diversity, and Inclusion organized in the second workshop on Language Technology for Equality, Diversity and Inclusion held as a part of the ACL 2022 (Chakravarthi et la., 2022).

**Table 6:** Original dataset statistics

Class	# tweets	%
HS	825	25.88
NHS	1301	40.81
None	1062	33.31
Total	3188	100.00
HS + NHS	2126

**Table 7:** Balanced dataset statistics

Class	# tweets	%
HS	825	50
NHS	825	50
Total	1650	100

### Experiments and Results

Of the total 1,650 tweets in the corpus, 60% were randomly selected for the training phase (990 tweets), 20% for validation and parameter tuning (330 tweets) and the remaining 20% for testing (330 tweets).

The traditional machine-learning algorithms involved in this study are:

- *Support Vector Machines (SVM)*. It is a classification algorithm that calculates the best hyper-plane that discerns among a set of classes (Cortes & Vapnik, 1995). SVM was widely adapted to solve classification problems, as stated in Fernández-Delgado et al. (2014).
- *Multinomial Naïve Bayes (MNB)*. It is a classification model suitable for discrete features, such as CountVectorizer or TF–IDF. This classifier is usually used as a baseline (Xu et al., 2017).
- *Logistic Regression (LR)*. It is a model that calculates the probability of the default class (Wright, 1995). It is based on a sigmoid function, with a S-shaped curve that can adjust to any real value between 0 and 1. This model is usually applied in binary classification. It is useful if in the dataset the labels are linearly separable. This model is usually adopted as baseline; however, other models such as SVMs are less susceptible to noise and outliers.

Besides, we have explored the following deep-learning architectures:

- *MultiLayer Perceptron (MLP)*. They are a simple deep-learning structure, composed by an input layer, an output layer, and a set of interconnect deep-learning layers composed by different neurons (Riedmiller, 1994). The interconnection among these layers are by means of activation functions. Due to its simple architecture, they do not exploit any of the properties of natural language.
- *Convolutional Neural Network (CNN)*. They are deep-learning networks that use pooling layers to generate high-order features. That is, CNNs exploit the spatial dimension of human language, as they cluster joint words that may convey different meanings from the meaning of each word in isolation. Therefore, CNN are useful for handling linguistic phenomena such as polysemy. It is worth noting that, in their origins, CNNs were used in computer vision, but they were applied later to solve supervised classification tasks (Lopez and Kalita, 2017).
- *Bidirectional Long Short Term Memory (BiLSTM)*. These kinds of networks take advantage of the temporal dimension of human language, as they consider the word order. Moreover, bidirectional recurrent networks can read text from left to right and vice versa (Gers et al., 2000).

#### Feature sets

The feature sets involved in this study are:

- *Term Frequency-Inverse Document Frequency (TF-IDF)*. This measure ponders the use of a particular word within a set of documents. It is well-known by the Information Retrieval research community and was presented in (Salton & McGill, 1983).
- *Term Frequency (TF)*. This represents the absolute number of occurrences of a term in a document.
- *Binary Term Occurrences (BTO)*. This is another weighting scheme we used within the bag-of-words representation where each term receives 1 if it is present in the document or 0 other-wise.
- *N-Grams features (NG)*. N-Grams features counts the occurrence of terms within a corpus. We apply the TF-IDF strategy in order to dismiss those terms that appear commonly in all documents as happens with stop-words. N-gram features have two well-known drawbacks. On the one hand, they are weak against linguistic phenomena such as homonymy or polisemy, mainly due as they do not take into account the context of a word (that is, others words surrounding the target word). To solve this drawback, we do not limit to extract unigrams but bigrams, and trigrams combined with character n-grams (with length between 2 and 7). On the other hand, n-gram features result in sparse vectors with several columns to 0. To solve this drawback, we reduced the n-grams by using Latent Semantic Analysis (LSA), resulting in a vector of length 100.
- *Linguistic features (LF)*. Linguistic features are those that captures different traits of human language and how people uses language to communicate ideas. In particular, we use the the UMUTextStats tool (García-Díaz et la., 2020; García-Díaz et al., 2021) to capture a wide variety of linguistic features organised in the following categories: (1) Phonetics, (2) Morphosyntax, (3) Correction and style, (4) Semantics, (5) Pragmatics, (6) Stylometry, (7) Lexis, (8) Psycho-linguistic process, (9) Register, and (10) Social media. It is worth mentioning that the linguistic features are both extracted from the documents and a cleaned version of them, in which the texts are lowercased, and some common errors are corrected using the PSpell library. Thus, features concerning correction and style are extracted from the original version of the text whereas other features are extracted from the cleaned version. Finally, as there are features that counts raw occurrences whereas others calculate percentages, we apply a MinMax scaler to normalize all features in a range of [0-1] and we apply a feature selection process to discard features strongly correlated.
- *Word Embeddings (WE)*. Embeddings is a technique to encode linguistic units (sentences, words, or characters among others) employing dense vectors. The core idea is that semantically similar units are clustered together. Specifically, we evaluate three techniques of word embeddings and two more deep-learning architectures. The word embeddings evaluated are: (1) word2vec (Mikolov et al., 2013), (2) gloVe (Pennington et al., 2014), and (3) fastText (Mikolov et al., 2018). The deep-learning architectures included are: (1) Convolutional Neural Networks (CNN), that are capable of exploiting spatial information of the texts, and (2) recurrent neural networks, that are capable of exploiting temporal information of the texts. Particularly, we evaluated Bidirectional Long-Short Term Memory (BiLSTM).
- *Sentence Embeddings (SE)*. We extract sentence embeddings from the texts using the Spanish pre-trained model from fastText (Grave et al., 2018), that encodes each document of the corpus as a vector of size 300. For this, fastText averages the word vectors of each document plus the EOS token.
- *Bert Embeddings (BE and BF)*. The last two feature sets evaluated are based on BERT (Devlin et al., 2018). Specifically, we use BETO, a Spanish version of BERT (Cañete et al., 2020). BERT is a bidirectional transformer capable of learn contextual embeddings from texts. It main advantage is that the codification of each word depends of its context, so it can handle synonymy and homonymy. In this work, we evaluate BERT embeddings from BETO applying two strategies: with and without fine-tuning the model. BERT embeddings without fine-tuning are BE. The idea is to the evaluate the BERT model as available, that is, in which word embeddings were trained from general purpose datasets. Next, we evaluated the model after fine-tuning (BF), in which the embeddings are adjusted to maximising the differences among hope speech. Although it is expect that the fine-tuned embeddings provide better results, we evaluated them separately to measure the impact of the adjustment process. To obtain both (BE and BF) we use the HuggingFace library to extract the embedding of the [CLS] token applying a mean pooling strategy on-top (Reimers and Gurevych, 2019).

#### Parameter setting

The traditional machine learning algorithms were implemented with the sklearn library and their configurations were as follows. For the SVM algorithm, the libSVM implementation was used and the formulation known as C-SVC with linear kernel and default configuration were selected. The term C in C-SVC refers to a regularization parameter that indicates to the algorithm if you need to prevent wrong classifications in each training example. Regarding Naïve Bayes, we used Naïve Bayes founded on a multinomial distribution, due to the length of the vocabulary, and default configuration. Finally, we tested regularized Logistic Regression with default configuration.

Regarding deep learning algorithms, for each feature set and architecture, we conducted an extra hyper-parameter optimisation stage with the validation dataset. All neural networks were trained with an early-stopping mechanism with a patience of 100. It is worth noting that these hyperparameters are selected because: 1) they are broad values that adapt to a large number of scenarios, 2) they have given good results in the past by our research group, 3) it is not clear which of these values are the optimum, except using a trial and error approach. Next, we detail the main hyperparameters evaluated.

- *Network type*. We evaluated different deep learning networks architectures ([MLP, CNN, BiLSTM]). The MLP is selected to evaluate the fixed feature sets, such as the linguistic features or the sentence embeddings. The CNNs and the BiLSTM were selected to evaluate, respectively, the spatial and temporal dimension of the pretrained word embeddings.
- *Network architecture*. All networks were evaluated with shallow neural networks that consist into one or two hidden layers with the same number of neurons per layer. However, in the case of MLP we also evaluated deep-learning models. These models have between 3 and 8 hidden layers. For deep-learning architectures, we arrange the number of neurons per layer in different shapes; namely, brick, funnel, rhombus, long funnel, diamond, or triangle).
- *Batch size*. We use mini-batch mode with two batch-sizes: 32, 64. The value of 32 is selected because it is a general default value.
- *Activation functions*. We evaluate the following activation functions: linear, selu, relu, elu, sigmoid, tanh. These activation functions were selected because they have different attributes that we want to explore, such as the vanishing gradient problem or saturation.
- *Learning rates*. We test two learning-rates: 10e–03, 10e–04; both with a learning rate scheduler based on time-decay. We usually prefer small learning rates as we have pretrained embeddings, but we will also test larger learning rates.
- *Dropout*. Dropout is a mechanism to prevent overfitting over the custom validation split. We evaluate three ratios (.1, .2, .3), and no dropout.

#### Results

Table 8 shows the results per feature set and architecture over the test split. We report the overall precision, recall, F1-score and accuracy of each combination. The first row corresponds to the baseline case, which achieved an F1-score = 78.47. The next eight results are those performed with traditional machine learning algorithms and with the different sets of features described above. We can observe that the results are similar, obtaining the best of these partial results with the combination of BTO and SVM (f1 = 79.05).

The last block of results corresponds to those obtained with deep learning algorithms. We highlight the best result, obtained with the BF (BETO fine-tuned) feature set, with a value of F1-score = 85.12, which is an 8% improvement over the baseline.

**Table 8:** Results

Feature set	Architecture	Precision	Recall	F1-Score	Accuracy
TFIDF	SVM (baseline)	78.51	78.48	78.47	78.48
TFIDF	MNB	77.67	77.57	77.55	77.57
TFIDF	LR	76.71	76.66	76.65	76.66
BTO	SVM	79.27	79.09	79.05	79.09
BTO	MNB	78.53	78.48	78.47	78.48
BTO	LR	78.80	78.78	78.78	78.78
TF	SVM	77.64	77.57	77.56	77.57
TF	MNB	78.19	78.18	78.17	78.18
TF	LR	78.21	78.18	78.17	78.18
NG	MLP	72.73	72.72	72.72	72.72
LF	MLP	77.01	76.06	75.84	76.06
SE	MLP	78.78	78.78	78.78	78.78
WE	MLP	80.11	80.00	79.98	80.00
WE	CNN	76.67	76.06	75.92	76.06
WE	BILSTM	75.38	74.54	74.33	74.54
BE	MLP	83.20	83.03	83.00	83.03
BF	MLP	**85.37**	**85.15**	**85.12**	**85.15**

We include in Table 9 the best hyperparameters for each feature set.

**Table 9:** Best hyperparameter selection for the neural network models

Feature set	Architecture	Shape	Layers	Neurons	Dropout	lr	Activation
NG	MLP	Brick	1	64	0.1	0.010	tanh
LF	MLP	Brick	2	128	0.3	0.001	Sigmoid
SE	MLP	Brick	1	2	False	0.001	Linear
WE	MLP	Brick	1	4	False	0.001	tanh
BF	MLP	Brick	2	8	0.2	0.001	relu

**Fig. 2:**
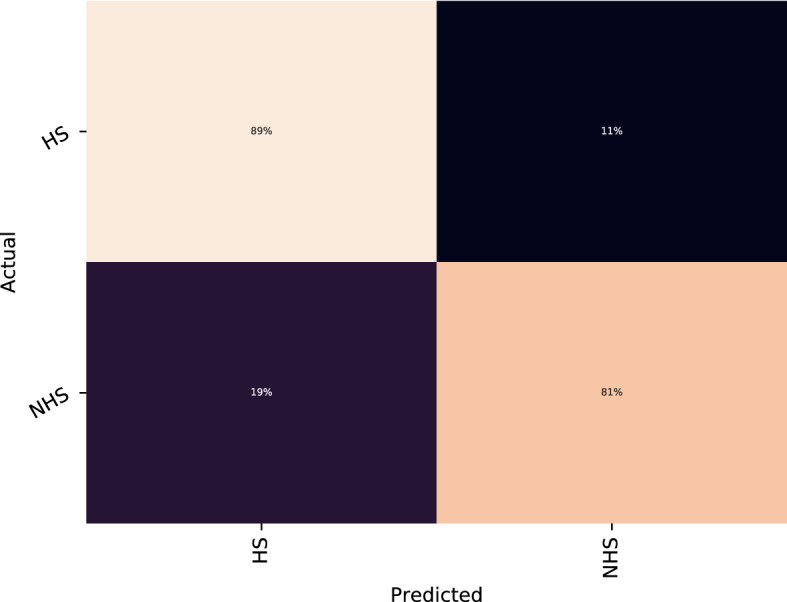
Confusion matrix of the best model (BF)

From Table 9, we reach the following insights:

- *Network architecture*. Best results are always achieved with shallow neural networks with one or two hidden layers. The reason all shapes are brick-shaped is because it is the only shape that is evaluated in shallow neural networks. Moreover, the best results with WE are achieved with MLP. Besides, the number of neurons is higher with linguistic and negation features; however, in networks based on word embeddings require fewer neurons to achieve good results.
- *Dropout mechanism*. The dropout mechanism varies. The non-contextual embeddings (regardless they are sentence or word embeddings) behave better without dropout. However, the linguistic features achieve better result with a dropout ratio of .3.
- *Learning rate*. All feature sets, except negation features, worked better with small values.
- *Activation function*. We observe different activation functions for each feature set. Tanh is the only one that appears twice in negation features and non-contextual word embeddings. However, as the number of hidden layers is small, we consider that this parameter is not very relevant.

In order to analyse the performance of the best model, we include the normalised confusion matrix of the BF model (see Figure 2). We can observe that the performance of both Hope Speech and Non Hope Speech labels are high. A 11% of the HS instances were incorrectly predicted as Non Hope Speech whereas a 19% of the Non Hope Speech labels were incorrectly predicted as Hope Speech.

### Discussion

The first step to analyze the results obtained was to get the hits and failures. Table 10 shows the total of hits and failures of the baseline case (bc) and the experiment that provided the best results (BF + MLP). We can observe a high percentage of hits and, if we focus on the failures, we can see that in both experiments they are more or less balanced. We consider that it is more important to solve the failures in which an NHS text is labeled as HS (Failures NHS in the table) than the opposite case (Failures HS in the table), because of the consequences that this could have.

**Table 10:** Hits and failures in the baseline experiment and the best experiment

Feature set	Architecture	Hits (HS)	Hits (NHS)	Failures (NHS)	Failures (HS)
TFIDF	SVM (bc)	137	124	41	28
BF	MLP	147	134	31	18

After a thorough analysis of the system errors, we have established a set of error categories, which are summarized in Table 11.

**Table 11:** Error categories summary

Category	Description
1.	Pretends promoting tolerance and integration of LGBT collective but hate speech vocabulary is used
1.1.	When 1. and writer uses uncommon or misspelled hate speech vocabulary, and/or complex expressions with the intent to demean and/or harass
1.2.	When 1. and writer uses uncommon or misspelled hate speech vocabulary, and/or complex expressions in a violent way
1.3.	On 1. and using LGBT collective frequent fixed expressions and/or vocabulary that out of that context could be interpreted as hate speech
2.	Annotator error
3.	Authors are in favor of LGBT collective integration but against any public claim of their rights
4.	Authors say not to be against the LGBT collective but deny its existence

The different errors detected are shown below, including an example in each case.

1. Pretends promoting tolerance and integration of LGBT collective but hate speech vocabulary is used, with three additional subcategories.
  1.1. Writer uses uncommon or misspelled hate speech vocabulary, and/or complex expressions with the intent to demean and/or harass. Rare hate speech vocabulary and/or expressions is used.

**Table:** 

Real class: NHS - Predicted class: HS	
@user Quiero pensar que es un giño al colectivo #LGTBIQ #LGTBI ya que en muchos campos muchas empresas homofobas se niegan a reconocer que muchos jugadores son homosexuales y en Canarias con lo ‘supuesta’ libertad hipócrita hay mucha homofobia.. conozco gente que lo oculta por miedo.	
*@user I want to believe that with this they want to reference #LGTBIQ #LGTBI collective, since in many areas numerous homophobic companies refuse to recognize that a lot of players are homosexual and in Canary Islands homophobia is very common.. I know people that hide it because of fear..*
  1.2. Writer uses uncommon or misspelled hate speech vocabulary, and/or complex expressions in a violent way.

**Table:** 

Real class: NHS - Predicted class: HS	
Mi bebida favorita todos los 28 de junio son las lágrimas frescas de fascistas.	

#Orgullo2021 #Orgullo #OrgulloLGTBI #OrgulloSiempre #OrgulloLTGBI	

*#2021Pride #Pride #LGTBIPride #ForeverPride #LTGBIPride*
  1.3. LGBT fixed expressions and/or vocabulary that out of context could be interpreted as hate speech.

**Table:** 

Real class: NHS - Predicted class: HS	
@user Yo creo que como tu miles de nosotros/as/es estamos en el mismo estado de ánimo. Se acabó el callar, disimular y mirar hacia adelante. NOS VAN A ENCONTRAR DE FRENTE, PREPARADOS Y DISPUESTOS A COMBATIR SU HOMOFOBIA. #LaRevoluciónDeLosMARICONES #Historia2021 #LGTBI #LGTBIfobia	
*@user I think that like you thousands of us are feeling the same.* *THEY WILL FIND US FACE TO FACE, READY AND WILLING TO FIGHT THEIR HOMOPHOBIA.* *#TheGAYRevolution #2021History #LGTBI #LGTBIphobia*
2. Annotation error. After the error analysis, some labeling errors have been detected. The annotators have been informed to make the corresponding revision of the dataset.

**Table:** 

Real class: NHS - Predicted class: HS	
Eres un vicioso. Mejor ser vicioso, liberal y poliamoroso que agredir al que no ame lo mismo que yoTengo más pero estas las que más. Y cada humillación, marginación, desprecios etc me ha hecho más fuerte cada día #Bisexual #Pride #Lesbianas, Gais, Bisexuales y Transgénero #Pride2021 #LGTBIQ #Orgullo2021	
*You are vicious. Better to be vicious, liberal and polyamorous than to attack the one who does not love the same as meI have more but these the most. And every humiliation, marginalization, slights etc has made me stronger every day #Bisexual #Pride #Lesbian, Gay, Bisexual and Transgender #Pride2021 #LGTBIQ #Pride2021.*
3. Authors say to be in favor of LGBT collective integration but against any public vindication of their rights.

**Table:** 

Real class: NHS - Predicted class: HS	
@user #Orgullo2021 #Orgullo #OrgulloLGTBI #OrgulloHetero ?‘Y el día del hetero? Basta de inventar días inveciles que solo buscan dividir aún más la sociedad, un día internacional de la variedad sexual o algo así, pero si te parece estúpido un día del hetero, lo mismo es tener día	
*@user #2021Pride #Pride #LGTBIPride #StraightPride* *Where is the Straight Day? Stop inventing stupid days only to divide the society even more, create an international day for the sexual variety or something like that, but if you think that having a Straight Day is stupid, it’s the same with*
4. Authors say not to be against the LGBT collective but deny its existence.

**Table:** 

Real class: NHS - Predicted class: HS	
@user Pero cuántas lamentaciones por los #LGTBI ellos no son ningún género diferentes, son varones y hembras que han escogido una maneras de vivir diferentes a como Dios lo creó, y Dios no crea seres imperfectos.	
*@user Don’t be so concerned about the #LGTBI they don’t have a different gender, they’re just men and women that have chosen to live in a different way, and God doesn’t create imperfect living beings.*

### ACL 2022 shared task on *hope speech* detection

The SpanishHopeEDI dataset presented in this work was used in the Shared Task on Hope Speech Detection for Equality, Diversity, and Inclusion organized in the second workshop on Language Technology for Equality, Diversity and Inclusion, held as a part of the ACL 2022^7^ (Chakravarthi et la., 2022). The participants were provided with annotated training and development sets, and unlabelled test sets in five different languages: Tamil, Malayalam, Kannada, English and Spanish. The goal of the shared task was to classify the given sentences into one of the following classes: *Hope speech* or *Non hope speech*. A total of 126 participants registered for the shared task and 14 teams finally submitted their results. The performance of the systems submitted were evaluated in terms of micro-F1 score and weighted-F1 score. The datasets for this challenge are openly available at the competition website^8^. As authors of the Spanish dataset, we are part of the organizing committee of the task as well.

## Conclusions and future work

In this article we perform a complete study on hope speech, analyzing existing solutions and available resources. In addition, we have generated a quality resource, SpanishHopeEDI, a new Spanish Twitter dataset on LGBT community for hope speech detection. Moreover, we have conducted some experiments that can serve as a baseline for further research.

As in HopeEDI (Chakravarthi, 2020) and KanHope (Hande et al., 2021) datasets, we proposed experimental methods for increasing the visibility of deprived communities as LGBT, while protecting the democratic right of freedom of speech not flagging for elimination any *hate speech* content and reinforcing instead *hope speech* comments. In addition, we find specially interesting and complementary the work from India-Pakistan dataset (Palakodety et al., 2019) about the hostility-defusing properties of *hope speech* and plan to continue researching on this approach.

Moreover, as initially we intended to continue the work from Chakravarthi (2020), then we created our first Spanish *hope speech* dataset. Nevertheless, and even though we obtained good results with an F1-score of 85.15, other authors obtained similar results with datasets (Hande et al., 2021; Mahajan et al., 2021) and therefore we consider important to keep researching about the quality of these datasets. As previously discussed, the SpanishHopeEDI dataset presented in this work was used in the LT-EDI Shared Task, held as a part of the ACL 2022(Chakravarthi et la., 2022).

As future work, we plan to deal with the semantic expression of sentences and to address the identification of *hope speech* in Spanish in different contexts in order to analyze the language characteristics of *hope speech*.

Taking into account that it is very demanding to generate and validate new datasets with this level of quality, manually labeled in a complete way, we will continue to increase and improve the presented herein, with this same subject and others related to EDI (Equality, Diversity, and Inclusion).

## Data Availability

The dataset, the annotation guideliness and the source code of the experiments performed are available at: https://github.com/Smolky/LREV-Hope-Speech-Detection-in-Spanish-2022

## Footnotes

1 https://itgetsbetter.org/.

2 https://github.com/philbot9/YouTube-comment-scraper.

3 https://huggingface.co/datasets/hope_edi.

4 In the HopeEDI dataset, the authors used an automatic language detector to filter out comments in English, Malayan and Tamil, but when they did the manual annotation they found that some of the comments that had been filtered out as being written in these languages really were not and decided to use the *Other Language* label for them.

5 https://huggingface.co/datasets/kan_hope.

6 The dataset, the annotation guideliness and the source code of the experiments performed can be found in the Availability of data and material section.

7 https://sites.google.com/view/lt-edi-2022/home

8 https://competitions.codalab.org/competitions/36393#learn_the_details-evaluation
